# Pulmonary Fibrosis and Diabetes Mellitus: Two coins with the same face

**DOI:** 10.26502/aimr.0165

**Published:** 2024-03-16

**Authors:** Yssel Mendoza Mari, Marcel P. Fraix, Devendra K. Agrawal

**Affiliations:** 1 Department of Translational Research, College of Osteopathic Medicine of the Pacific, Western University of Health Sciences, Pomona CA 91766

**Keywords:** Diabetes mellitus, Extracellular matrix, Idiopathic pulmonary fibrosis, Nintedanib

## Abstract

Idiopathic pulmonary fibrosis (IPF) constitutes a long-term disease with a complex pathophysiology composed of multiple molecular actors that lead to the deposition of extracellular matrix, the loss of pulmonary function and ultimately the patient’s death. Despite the approval of pirfenidone and nintedanib for the treatment of the disease, lung transplant is the only long-term solution to fully recover the respiratory capacity and gain quality of life. One of the risk factors for the development of IPF is the pre-existing condition of diabetes mellitus. Both, IPF and diabetes mellitus, share similar pathological damage mechanisms, including inflammation, endoplasmic reticulum stress, mitochondrial failure, oxidative stress, senescence and signaling from glycated proteins through receptors. In this critical review article, we provide information about this interrelationship, examining molecular mediators that play an essential role in both diseases and identify targets of interest for the development of potential drugs. We review the findings of clinical trials examining the progression of IPF and how novel molecules may be used to stop this process. The results highlight the importance of early detection and addressing multiple therapeutic targets simultaneously to achieve better therapeutic efficacy and potentially reverse lung fibrosis.

## 1. Introduction

Idiopathic pulmonary fibrosis (IPF) is a chronic, progressive fibrotic lung disease characterized by a restrictive ventilatory defect and impaired gas transfer due to deposition of fibrotic tissue in the lung interstitium [1]. The pathology of IPF is characterized by disruption of normal lung architecture due to the deposition of excessive collagen and extracellular matrix (ECM) in the alveolar walls and the aggregation of proliferating fibroblasts and myofibroblasts, which are recognized as fibroblastic foci on histologic evaluation [2]. IPF encompasses 17 to 37% of all interstitial lung disease diagnoses [3] and commonly affects patients over 60 years, which undergo progressive failure of lung function that causes death on average three years after diagnosis [4]. The etiology of IPF remains unclear, but growing evidence points towards complex interactions between genetic and environmental factors in the setting of age-associated disease processes [5,6]. This leads to alveolar injury, aberrant epithelial-fibroblast interactions and thickening of lung matrix, resulting in the remodeling of lung interstitium [1]. Treatment options for IPF are limited and are largely palliative. Two pharmaceutical agents, pirfenidone and nintedanib, are licensed as novel IPF treatments. However, these agents decrease the rate of decline in the lung function and risk of acute deterioration of lung function, rather than halt or reverse the fibrogenic process [7,8]. Lung transplantation is the only option that offers hope for long-term survival, but it is only available to very few individuals [9].

## 2. Initiation and Development of Fibrosis

After an injury, lung tissue triggers a physiological reparative response. During this healing process, there is an interaction of multiple cell types, including immune system cells, epithelial cells, and fibroblasts. Besides the activation of lung epithelium stem cells and fibroblasts, alveolar macrophages initiate a pro-inflammatory response leading to the clearance of an insult and triggering a healing process through the secretion of growth factors, including transforming growth factor β1 (TGF-β1) [10]. If the injury signals persist, inflammatory response increases, leading to the cellular release of cytokines such as tumor necrosis factor-α (TNF-α), interleukin-1β (IL-1β) and interleukin-8 (IL-8) and the recruitment of neutrophils, monocytes and T cells to the alveolar space. This ultimately leads to matrix deposition and the progression of fibrosis [10]. The unregulated expression of pro-fibrotic factors like TGF-β1 [11] results in the accumulation and trans-differentiation of fibroblasts into pro-secretory myofibroblast phenotype, α–smooth muscle actin (α-SMA)-positive, which leads to excessive deposition of ECM constituents [10]. Under physiological conditions, fibroblasts express different subtypes of collagen and elastin. In the correct proportion, tissues acquire different degrees of stiffness and/or flexibility. Fibronectin and laminin, also expressed by fibroblasts, facilitate the connection between cells and ECM and interact with transmembrane adhesion protein such as integrin [12]. The abnormal activation of lung fibroblasts constitutes a modulating factor after injury, tipping the balance to the development of fibrosis, instead of towards a physiological reparative response [13]. Besides the stimulation of ECM protein expression, TGF-β1 reduces the function of enzymes involved in ECM degradation such as collagenase and matrix metalloproteinases (MMP), thereby resulting in accumulated ECM deposition. Besides ECM degrading enzymes, fibroblasts of the fibrotic lung also express four types of tissue inhibitor of metalloproteinase (TIMPs) [14]. The result is an unevenness between MMPs and their inhibitors in favor of TIMPs, which explains the decrease in collagen degradation in the damaged parenchyma.

During the development of fibrosis, resident epithelial cells also trans-differentiate into myofibroblasts, in a process called epithelial-mesenchymal transition (EMT) [15]. Epithelial cells undergo cytoskeletal remodeling, lose their cell-cell adhesion markers, like E-cadherin, and acquire mesenchymal phenotype markers such as N-cadherin, vimentin and α-SMA, contributing to fibrosis progression [15]. EMT is mainly driven by TGF-β1, but also by some other extracellular ligands such as epidermal growth factor (EGF), fibroblast growth factor, connective tissue growth factor (CTGF), nuclear factor-κB (NF-kB), among others [15]. TGF-β1 mediates fibrous proliferative effects by inducing apoptosis in alveolar epithelial type I cells [16].

The investigation of miRNAs levels in fibrotic tissue in animal models and human disease has led to a better understanding the progression of fibrosis [17]. Some miRNAs are expressed only in fibroblastic foci, but not in whole IPF lung tissue nor cultured fibroblasts [18]. More recently, the combination of laser capture microdissection and next-generation sequencing in fibroblastic foci identified key miRNA molecules, including miR-370–3p, miR-222–3p, miR-146a-5p, and miR-203a-3p. These miRNAs were previously described only in liver fibrosis [19]. In addition, miR-4454 and miR-23a-3p were found to be involved in cardiac and renal fibrosis [20, 21].

Another biological process involved in the progression of pulmonary fibrosis is endoplasmic reticulum stress (ER stress). The accumulation of unfolded or misfolded proteins activates a signal response termed unfolded protein response [22]. ER stress in lung is involved in severe damage to epithelial cells [1], fibroblast proliferation and myofibroblast differentiation [23]. ER stress inhibitors reduce EMT in animal models of pulmonary fibrosis and fibroblast proliferation; this last mechanism specifically regulated through the modulation of the PI3K/AKT/mTOR signaling pathway [24].

Cellular senescence is another key process during fibrosis establishment and evolution. The senescent phenotype is characterized by two major features: loss of cell division capacity and secretion of a large number of mediators known as senescence-associated secretory phenotype (SASP) [25]. Senescent cells do not respond to mitogenic stimuli, but rather undergo growth arrest and are positively identified by the expression of senescence associated-β-galactosidase (SA-β-gal) [25]. The SASP pattern of expression includes multiple growth factors, cytokines, chemokines, and matrix remodeling proteases such as TGF-β1, CTGF, TNF-α, IL-1α/β, IL-6, IL-8, ICAM, MCP1, MMP2, MMP3, MMP9, [26]. Accordingly, senescent cells induce the development of a pro-fibrotic and pro-inflammatory local environment, gradually affecting the whole organ [27]. In lungs, alveolar epithelial cells type II can become senescent, losing their capacity to regenerate alveolar epithelial cells type I lost by external injuries. SASP attributable to these cells triggers trans-differentiation of fibroblasts into myofibroblasts, amplifying the risk of developing pulmonary fibrosis [28]. But fibroblasts per se also become senescent, and express high levels of SA-β-gal and cell cycle inhibitors like P16, P21 and P53 [29]. Mitochondrial dysfunction, telomere shortening, epigenetic modifications, DNA damage, protein homeostatic imbalance and decreased autophagy are among factors involved in IPF [30]. Inhibition of autophagy has been related to accelerated senescence in epithelial cells, enhanced EMT and fibrosis [31]. Besides, lack of autophagy in lung fibroblasts may promote their trans-differentiation into myofibroblast phenotype through cellular mechanisms involving the inhibition of beclin-1 [32], caveolin-1 [33] or mTOR signaling pathway [34].

Development of fibrosis leads to a failure of physiological lung function characterized by insufficient gas exchange and interruption of oxygen supply [35]. Although hypoxia could be considered a result of fibrotic induration, hypoxic signals could trigger feedback loops during in the pathogenesis of the disease, perpetuating the fibrotic state through the stimulation of myofibroblast differentiation, ECM deposition and cell cycle modification [36]. In this regard, hypoxia-inducible transcription factors (HIF-1α and HIF-2α) play central roles by controlling the expression of a myriad of genes during acute or chronic hypoxia [37].

A recent concept classifies IPF as a metabolic disease, in which abnormal lipid levels have been quantified in serum and bronchoalveolar lavage fluids of patients [38]. It has been shown that high fat diets could increase the risk of developing pulmonary fibrosis [39]. A recent study showed that high-fat and high-fructose diet induced lung fibroblasts inflammation through transcriptional up-regulation of dedicator of cytokinesis 2 molecule, facilitating fibrotic progression [40]. Elevated cholesterol levels in serum potentiate lipid accumulation in alveolar epithelial cells and up-regulation of toll-like receptor 4/NF-κB signaling, leading to low grade pulmonary inflammation and fibrosis [41]. High lipidic levels also affect normal epithelial stem cells proliferation, decrease mitochondrial b-oxidation capacity, and induce M1 macrophage polarization, all of this contributing to pave the way for lung fibrosis development [42].

## 3. Diabetes Mellitus and IPF

**Figure 1:**
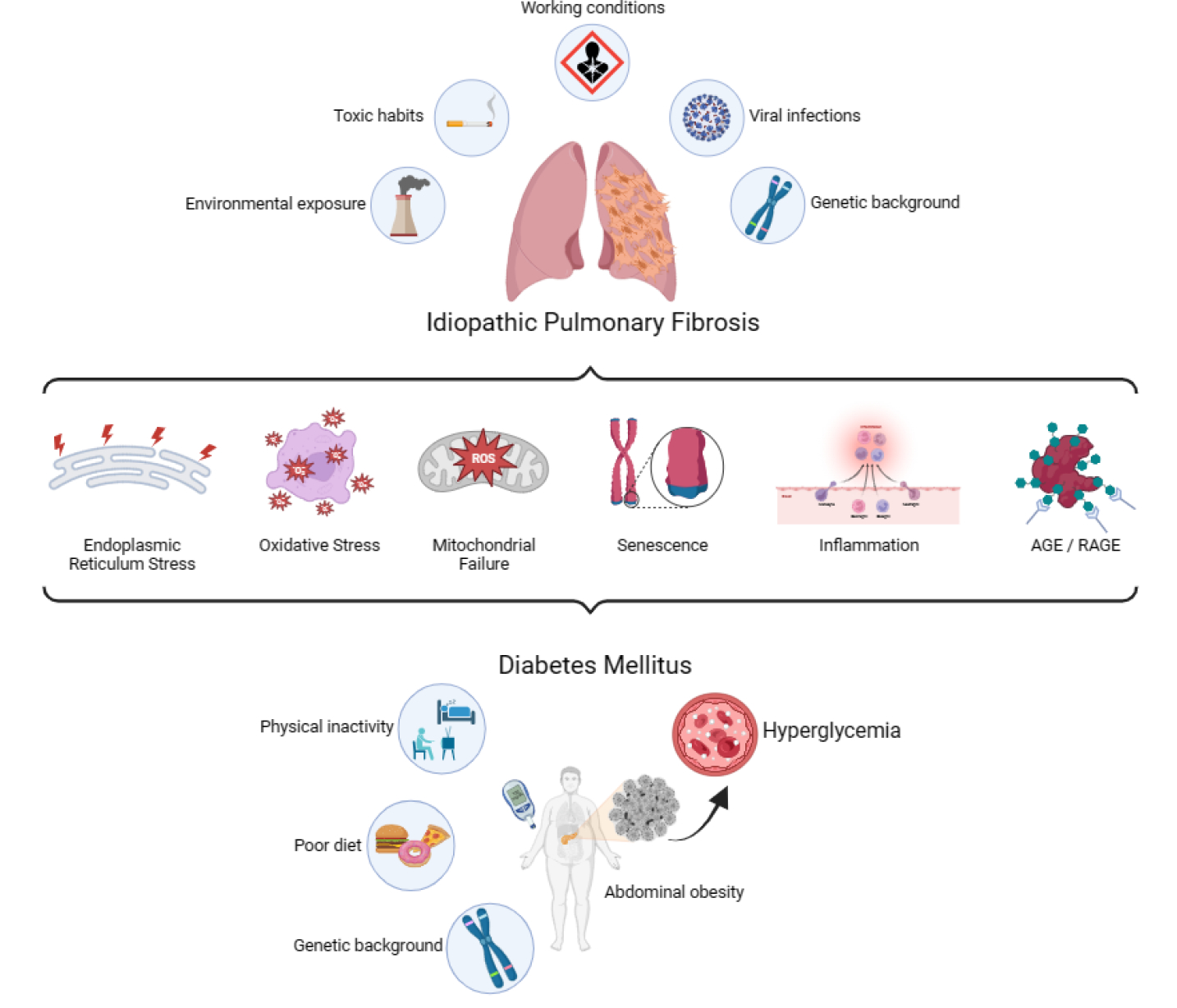
Common underlying molecular mechanisms of Idiopathic Pulmonary Fibrosis and Diabetes Mellitus.

Diabetes mellitus (DM) is a group of metabolic disorders in which genetic susceptibility is associated with environmental factors. More specifically, phenotypic expression results from the interaction of genes and the environment [43]. One of the main consequences of DM is the development of long-term vascular complications, due in part to chronic hyperglycemia, which causes damage to the blood vessels (angiopathy). Diabetic complications are classified into microvascular diseases, when the damaged vessels are of small caliber, and macrovascular diseases, when the arteries are compromised. Microvascular complications typically include retinopathy, nephropathy, and neuropathy. Macrovascular diseases include cardiovascular diseases, which can result in myocardial infarction, and cerebrovascular diseases, which lead to stroke [44]. In the last decades, increasing evidence corroborates the lung as another target organ for diabetic complications [45]. Just as sustained hyperglycemia is considered the “foundational stone” in the development of diabetic complications, there is experimental evidence indicating its involvement on a molecular level for development of pulmonary fibrosis in patients with DM [46]. Both clinical entities share numerous characteristics and molecular intermediaries that contribute to symptoms and reduce the quality of life in patients. Among these features are inflammation [47], ER stress [24], senescence [48], endothelial and mitochondrial dysfunction [49], oxidative stress [50], failure in tissue repair mechanisms [51], non-enzymatic glycation [52], excessive expression of proteases [53], and increased expression of TGF-β1 [54], all of which are driven and finely tuned by epigenetic mechanisms and a strong interaction of the organism with its environment (Figure 1). Previously, there were contradictory results regarding the association between IPF and DM [55], but a recent meta-analysis conducted by Bai and colleagues [56] demonstrated a positive correlation between both pathologies, although they could not find the specific causal relationship.

IPF patients present with a higher prevalence of DM compared to people with other lung diseases or healthy individuals [57]. Particularly, hyperglycemia is actively associated with pulmonary fibrosis [46]. It has been demonstrated that glucose burden might cause interstitial fibrotic alterations and alveolar microangiopathy [58] by increasing oxidative stress mediators, endothelial and immune cells activation and secretion of pro-inflammatory and pro-fibrotic cytokines [47]. Among other factors, the exacerbated generation of reactive oxygen species (ROS) by mitochondria contributes to explaining the development of microvascular complications in DM [59]. Also, in IPF lungs, mitochondrial ROS production is increased, and these levels can activate inflammatory mediators like (NF-kB) [60].

In lungs of IPF patients there is a downregulation of key enzymes involved in glycolysis, mitochondrial β-oxidation and tricarboxylic acid cycle [61], the same phenomenon that is observed in granulation tissue from diabetic foot ulcers [62]. From a histological point of view, pulmonary capillaries show thickening of the basal lamina [63], also characteristic of ischemic and neuropathic diabetic foot ulcers, which exhibit arteriolar wall thickening and dense fibrotic matrix infiltrated with inflammatory cells [64]. Several molecular mediators of glucose metabolism have been implicated in the fibrosis of lung tissue. For example, aerobic glycolysis plays a role during the pathological activation of lung fibroblasts, contributing to the progression to a fibrotic state [65] and also activates the YAP–TAZ signaling pathway. One of the most important transcription factors of CTGF [66]. CTGF is also an essential mediator of ECM protein expression in response to hyperglycemia, as well as TGF- β1 [67] and regulates glucose uptake in fibroblastic foci as a fuel to maintain ECM accumulation and fibrotic lesions. Among the enzymes involved in aerobic glycolysis, 6-phosphofructo-2-kinase/fructose-2, 6-biphosphatase 3 (PFKFB3) is required for the initiation and sustainment of myofibroblast differentiation [68] and has been implicated in the ECM production by lung fibroblasts [69]. It has been shown that the downregulation of PFKFB3-associated aerobic glycolysis decreases collagen synthesis in lung fibroblasts challenged with LPS via regulating the AMP-activated protein kinase (AMPK)/mammalian target of rapamycin pathway [70].

Also, GLUT1-dependent glycolysis regulates the activation of fibrogenesis in aged lungs *in vivo and in vitro* [65]. This glucose transporter is highly conserved in mammalian cells [71] and, among other mechanisms, is regulated by the STAT3/p-STAT3 signaling pathway [72]. Previous studies have demonstrated that GLUT1 [73] and STAT3/p-STAT3 signaling pathway [74] are involved in the progression of fibrosis. IPF-derived lung fibroblasts express higher levels of GLUT1 compared to normal lung fibroblasts. In an equivalent way, GLUT1 is significantly increased in IPF patient’s lung tissues, mainly in FF, compared to the lungs of control subjects [75]. It has been demonstrated that the pharmacological inhibition of GLUT1 diminishes the expression of α-SMA in primary fibroblasts, through the modulation of STAT3/p-STAT3 signaling pathway [75] or through the activation of AMPK [65]. AMPK activation also diminishes the expression of miR-27a [76], a potent inhibitor of peroxisome proliferator-activated receptor gamma (PPARG), which is necessary to decrease the pro-fibrotic state.

Another common feature between IPF and DM is the deregulation in lipid metabolism. Lipid metabolism is associated with glucose metabolism, as acetyl-CoA can be converted into lipids. Lysophosphatidic acid (LPA) and sphingosine 1-phosphate (S1P) are highly involved in the differentiation of fibroblasts to myofibroblasts and EMT pathways [77], TGF-β1 activation, prevention of apoptosis in fibroblasts, induction of epithelial apoptosis, and increase of vascular permeability [78]. Inhibition of S1P diminishes the expression of CTGF, leading to the amelioration of fibrosis [77]. Different metabolomic studies have shown an accumulation of circulating free fatty acids (FFAs) in IPF lung tissue, plasma, and bronchoalveolar lavage fluid of IPF patients, and have been found to correlate with the disease progression and outcome [61]. High levels of FFAs may influence pulmonary fibrosis by regulating the TGF-β1-induced activation and proliferation of fibroblasts. It also has been indicated that alterations in the FFAs metabolism contribute to epithelial ER stress, apoptosis, EMT, and M2 polarization [79], all of them crucial elements during the IPF development.

## 4. Key Factors in the Development of Fibrosis

### 4.1 Connective Tissue Growth Factor (CTGF, CCN2)

**Figure 2:**
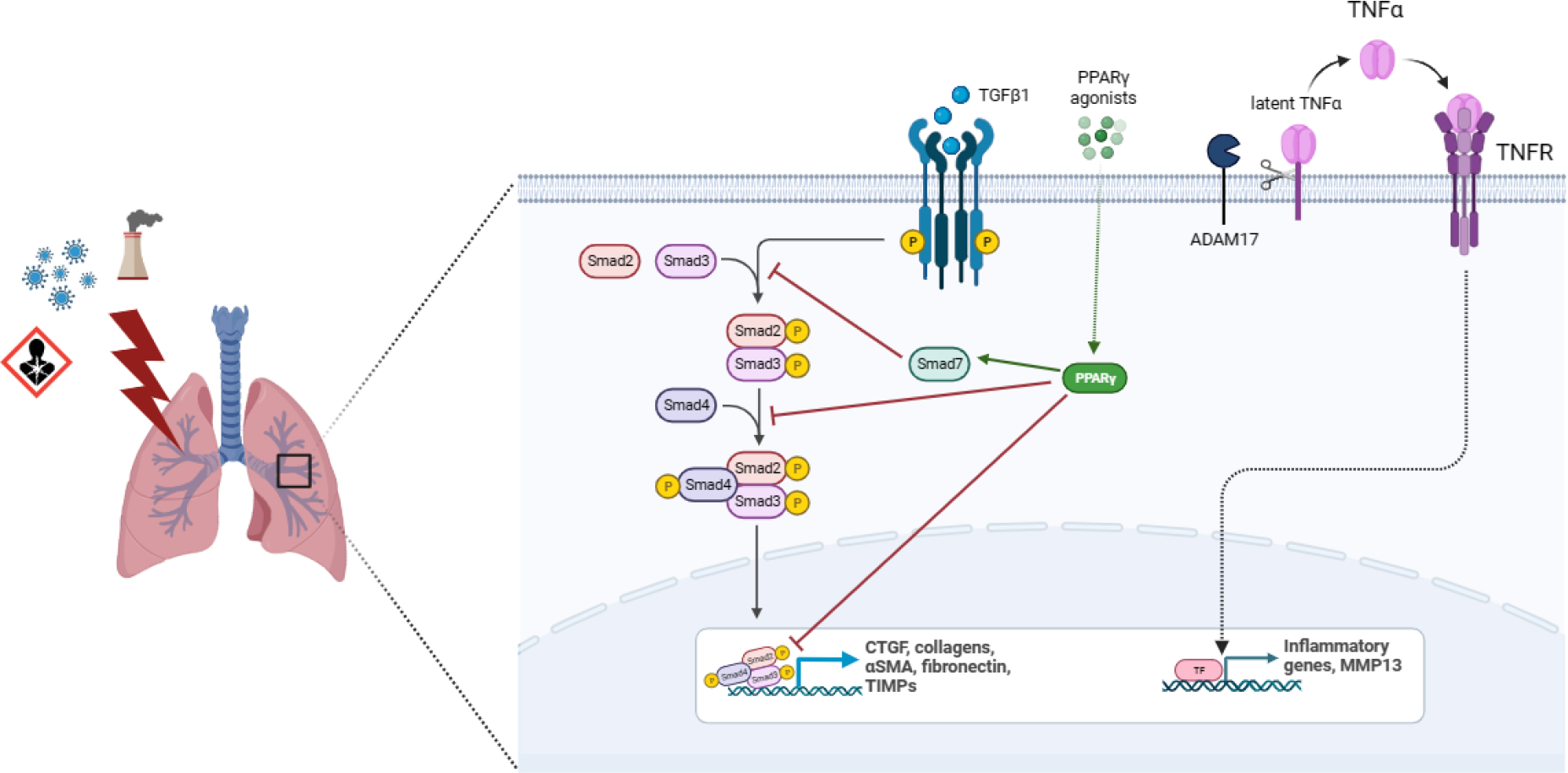
Key factors in the pathophysiology of Idiopathic Pulmonary Fibrosis. Connective tissue growth factor (CTGF), peroxisome proliferator-activated receptor gamma (PPARG), matrix metalloprotease 13 (MMP13) and A disintegrin and metalloproteinase 17 (ADAM17).

Another molecule highly implicated in fibrosis progression is CTGF (Figure 2). This protein is induced by and acts downstream of TGF-β1, potentiating its profibrotic activity. After binding to specific receptors, CTGF regulates the availability and activity of several cytokines and mediates the matrix turnover by binding to ECM proteins [80]. CTGF is expressed in mesenchymal cells and mediates physiological tissue regeneration and pathological fibrosis via ECM deposition, fibroblast proliferation, matrix production, angiogenesis, and granulation tissue formation [81]. This growth factor was found to be upregulated in bronchoalveolar lavage, lung tissue and plasma from IPF patients as well as in cultured fibroblasts [82]. CTGF is secreted by alveolar epithelial cells type II and activated fibroblasts in an autocrine and paracrine ways, promoting EMT and fibroblasts migration and proliferation, through the activation of several molecular pathways involving PI3K and ILK [83]. M2 macrophages also secrete CTGF to reinforce fibroblast proliferation, migration, adhesion, and ECM production via activating the AKT–ERK1/2–STAT3 pathway [84]. CTGF also plays important roles in different mechanisms involved in fibrosis such as fibrocyte differentiation, senescence, glucose, and glutamine metabolism [80,81]. It has been demonstrated that CTGF deletion diminished COL1, COL3 and fibronectin expression, contributing to attenuating experimentally induced pulmonary fibrosis and pulmonary arterial hypertension [85]. Besides, neutralization of CTGF by pamrevlumab, a specific anti-CTGF monoclonal antibody, suppress TGF-β1-induced fibroblast proliferation and myofibroblast differentiation and mesothelial to mesenchymal transition in IPF [86].

Besides its implication in lung fibrosis, CTGF also plays an active role in DM complications, especially in diabetic retinopathy, in which promotes thickening of the retinal capillary basal layer and pericytes loss. Besides, CTGF stimulates growth of endothelial cells, adhesion, and ECM deposition in diabetic retinas, and it is induced by AGE and growth factors such as VEGF [87]. Lack of CTGF allele in mice with long term DM reduces the perithelial cell and acellular capillary generation and controls the thickening of the retinal capillary basement membrane [88]. CTGF is also related to diabetic nephropathy. It was found in glomerular cells, tubular epithelial cells, and interstitial cells of the diabetic kidneys and was upregulated in glomeruli of streptozotocin-induced diabetic rats and in primary human mesangial cells stimulated by glucose [89]. CTGF increased the tissue inhibitor of matrix metalloproteinases 1 (TIMP-1) expression in diabetes, preventing matrix degradation and stimulated EMT in renal tubular cells in diabetes, leading to genesis of new fibroblasts in the renal interstitium [90]. It has been shown that blocking CTGF not only attenuated its effects associated to fibrotic process, but also decreased proteinuria, albuminuria, and serum creatinine [91].

### 4.2 Peroxisome Proliferator-Activated Receptor Gamma (PPARG)

Peroxisome proliferator-activated receptors are ligand-activated transcription factors of the nuclear receptor superfamily that regulate metabolic homeostasis of the cell. Among them, PPARG is well known to regulate synthetic metabolism (anabolism) in the adipose tissue and plays a key role in glucose and lipid metabolism, insulin sensitivity, and inflammation [92]. PPARG is broadly expressed in various cell types, including adipocytes, lung epithelia, fibroblasts, and inflammatory macrophages, and is essential for lung homeostasis [93]. In isolated human or mouse lung fibroblasts, PPARG silencing potentiates profibrotic phenotypes [94]. Post-transcriptional regulation of PPARG by microRNAs is implicated in different diseases. Specifically, PPARG downregulation by miR-27a leads to the activation of TGFb/Smad3 signaling cascade and further development of fibrosis in kidney [95]. It has been shown that hyperglycemia also decreases PPARG activity through an upregulation of miR-27a [96]. This miRNA has shown high expression levels in DM and is positively correlated with fasting glucose levels in patients with type 2 diabetes [97]. Another element of epigenetic control is genomic DNA methylation. Gene expression studies have revealed noticeable differences in the transcriptional state in the lung parenchyma of IPF patients, compared with those in normal individuals [98]. Besides, there is a strong association between DNA methylation levels and expression of numerous fibrogenic genes in mouse fibrotic lung tissues or lung fibroblasts of IPF patients [99], suggesting an active role of this epigenetic modification during the initiation and progression of IPF. In the case of PPARG, previous studies have demonstrated that promoter hypermethylation is associated with liver fibrosis, osteoarthritis, diabetes, and atherosclerosis [100]. Specifically, in IPF patients, an increase in DNA methyltransferases DNMT1 and DNMT3a activity has been associated to an hypermethylation of PPARG promoter [101].

The potential antifibrotic effect of PPARG has been demonstrated in several experimental models. Activation of PPARG by agonists attenuates fibrosis in kidneys [102], liver [103], heart, [104] and lungs [105]. Recent results showed that a PPARG agonist inhibited the expression of TGF-β1, fibronectin and collagen-I after restoring levels of PPARG in a lung fibrosis model induced by silica exposure in mice [106]. It is well established that PPARG inhibits collagen synthesis at transcriptional level [107] and may alter connective tissue target genes by blocking TGF-β1 signaling [108] (Figure 2). PPARG knockdown has been associated with reduced PPARG cofactor 1 alpha and with stimulating mitochondrial fragmentation and superoxide production [109] and its activation prevents high glucose-induced increases in TGF-β1 expression [110]. Besides, PPARG can beneficially and directly regulate the expression of antioxidant enzymes [111], contributing to decrease the excessive levels of ROS, molecules directly involved in the development of pulmonary fibrosis. Encouraging results using PPARG agonists might be used as a starting point to carry out new investigations that lead to the development of highly effective drugs that potentiate the control of fibrosis and metabolic imbalance in those patients who require it, for example DM-IPF individuals.

### 4.3 Matrix Metalloproteinase-13 (MMP13)

One of the key features of fibrosis is an excessive deposition of ECM proteins in compromised organs, being the imbalance of MMPs and their TIMPs as one of the elements that significantly contribute to this pathogenic state [112]. MMPs are a family of inducible, zinc-dependent, secreted or cell surface endopeptidases that are centrally involved in the dynamic of ECM. Expression of MMPs and their physiological inhibitors TIMPs, is tightly regulated in the lung, with notable activity during lung development, tissue injury, and host defense [113]. MMPs have been highly implicated in fibrosis. Patients with IPF have shown increased levels of MMP1, 3, 7, 8, and 9, being MMP7 substantially associated with greater severity, worsening, and short survival time [114].

MMP13, also known as collagenase 3, is the principal interstitial collagenase and has a high specificity for degrading insoluble fibrillar collagens, especially type II and I. MMP13 was shown to be significantly upregulated at the mRNA and protein level in IPF lungs in a study where 16 patients were enrolled [115]. This result was corroborated in the multicenter observational US Idiopathic Pulmonary Fibrosis Prospective Outcomes (IPF-PRO, Registry NCT01915511), in a cohort of 300 patients [116]. In this study, circulating levels of MMP13 were directly associated to a reduction on diffusing capacity of the lungs for carbon monoxide and composite physiologic index [117], indicators for disease severity which correlates with the extent of fibrosis on radiography in patients with IPF [118]. However, in animal models the scenario is different. MMP13 has been shown to be downregulated in a model of pulmonary fibrosis induced in rats with paraquat and hyperoxia [119]. Also, in a murine model of bleomycin-induced lung fibrosis, MMP13−/− mice exhibited an increased inflammatory reaction and a greater extent of fibrosis compared with wild-type animals [115], but in a murine model of radiation-induced pulmonary fibrosis, MMP13 reduced pulmonary inflammation and fibrosis [120]. It is known that MMP13 cleaves CCL2 and CXCL12, reducing their activity [121]. This suggests that increasing the levels of MMP13 could induce an anti-fibrotic effect by decreasing the recruitment of CCR2-expressing “profibrotic” macrophages [122] and CCR2- and CXCR4-expressing fibrocytes [123]. According to these results, MMP13 is considered as an anti-fibrotic MMP, and increasing its levels may have therapeutic effect in IPF clinical setup. In any case, the effectiveness of MMP13 stimulation or inhibition must be very exhaustively verified, given its pivotal role (Figure 2).

In diabetes, serum MMP13 was not different in ulcerated diabetic patients compared to healthy control individuals [124], but a focal increase in MMP-13 expression was observed in atherosclerosis [125]. In diabetic retinopathy, the activation of the runt-related transcription factor 2 pathway highlighted MMP13 as one of the putative target proteins for this diabetic complication. A different study demonstrated that elevated levels of MMP-13 in human monocytes were associated with hyperglycemic conditions, suggesting that this enzyme might contribute to diabetic retinopathy through its action in myeloid cells [126]. Glyburide, a hypoglycemic drug, has shown direct inhibitory effects on many metalloproteinases, including MMP-13 [127], suggesting that this mechanism could contribute to minimizing the damage observed in the retina due to the high glucose burden. Further studies should be carried out in animal models to investigate the role of this MMP in the diabetic context.

### 4.4 ADAM17

A disintegrin and metalloproteinase 17 (ADAM17), also known as TNF-α converting enzyme (TACE), is a type I transmembrane protein belonging to the adamalysin subfamily of Zn-dependent metalloproteases with the ability to cleave cell surface proteins, such as TNF-α, TGF-α and EGF receptor (Figure 2). Release of these cell-surface proteins to the extracellular space impacts cell adhesion, cell-cell interactions, and inflammatory responses [128]. Elevated levels of ADAM17 promote an increase of soluble IL-6 receptor α in the lungs, contributing to the development and progression of pulmonary fibrosis [129]. Another study showed that this enzyme is involved in hypoxia-stimulated CTGF expression in human lung fibroblasts WI-38 and the addition of an ADAM 17 inhibitor to these cells reduces the expression of CTGF [130].

In diabetes context, it has been demonstrated that the ADAM17 inhibitor JTP-96193 reduced TNF-α release from the fat tissue, prevented development of diabetes, and improved insulin resistance in mouse models of obesity and diabetes respectively. This molecule also prevented the delay of sciatic motor nerve conduction velocity in STZ-induced diabetic mice [131], contributing to diminish peripheral neuropathy associated to diabetes. The interplay between inactive rhomboid protein 2 (iRHOM2) and ADAM17 have been extensively studied due to iRHOM2 activity in promoting ADAM17 trafficking, maturation, and activity from the endoplasmic reticulum to the Golgi [132]. Some natural compounds such as anemonin proved to reduce the level of pro-inflammatory cytokines, ROS, iRhom-2, TACE, TNF-α, and inducible nitric oxide synthase expression in a streptozotocin-induced diabetic nephropathy in rats [133]. Another substance, diosgenin, has been shown to reduce dyslipidemia, hypertension, and pro-inflammatory cytokines (TNF-α, IL-1β, and IL-6) in the aorta of diabetic animals through modulating the iRhom2/TACE signaling molecules [134]. Other studies have revealed the effect of this compound in reducing obesity-induced systemic and local adipose inflammation, apoptotic proteins, and oxidative stress in the pancreas and adipose tissue of T2DM rats via modulating the ER stress-induced iRhom2/TACE signaling pathway [135].

## 5. Anti-fibrotic Therapies

### 5.1 Approved drugs

In October 2014, the FDA approved pirfenidone and nintedanib for treating IPF [136]. Pirfenidone is a synthetic small-molecule derivative of pyridone that improves fibrosis, inflammatory responses, and oxidative stress [137]. According to clinical trials CAPACITY I, CAPACITY II [138] and ASCEND [7], pirfenidone reduced the mean decline in forced vital capacity (FVC) percent predicted over 72 weeks compared with placebo. The treatment was also associated with decreased all-cause mortality and IPF-specific mortality [7]. The patients receiving pirfenidone also had a lower risk of respiratory related hospital admissions [139]. Pirfenidone exerts its effect by inhibiting different mechanisms that contribute to the development of fibrosis in the lung (for a review see [140]): attenuates fibroblast proliferation, myofibroblast differentiation, collagen synthesis, fibronectin production, and deposition of ECM by inhibiting fibrogenic growth factors, specially TGF-β1 [141] and one of its canonical signaling pathways [142]; diminishes the production of cytokines and accumulation of inflammatory cells [143] and regulates and reduces oxidative stress markers in the lung [144].

The approval of nintedanib by the FDA was based on two INPULSIS phase 2 clinical trials [8], in which the drug proved to reduce the annual rate of decline in FVC of the lungs at week 52 compared to placebo. Nintedanib is an intracellular tyrosine kinase inhibitor, originally developed as an anti-angiogenic cancer drug that binds and blocks receptors for platelet derived growth factor, fibroblast growth factor, and vascular endothelial growth factor [145]. The inhibition of these growth factors signaling reduces the proliferation and migration of lung fibroblasts, their trans-differentiation into myofibroblasts and the deposition of ECM [145].

The combined use of pirfenidone and nintedanib was assessed in several clinical trials. The addition of nintedanib to an ongoing pirfenidone therapy proved to be safe in a randomized phase 2 study in Japanese patients with IPF [146] and in a later single-arm, open-label, 24-week study [147]. The inverse combination was also evaluated in a trial in which IPF patients, who completed 4 to 5 weeks of nintedanib with no interruption or dose reduction, were randomized to receive nintedanib with add-on pirfenidone or nintedanib alone in an open-label study for 12 weeks. Both arms of this study showed good safety and tolerability, with only the known adverse events described for each drug [148].

Although both drugs contribute to slow the progression of IPF, they do not reverse the fibrotic state, being lung transplantation the only definite cure. Besides, there are some adverse effects that limit their use. For example, pirfenidone might provoke gastrointestinal symptoms, skin rashes, and photosensitivity [149] and can cause serious liver function abnormalities in 5% of patients, that is why regular monitoring is recommended [7].

The leading international societies on the management of IPF published a unified clinical practice guideline in which nintedanib is suggested for the treatment of progressive pulmonary fibrosis in patients who have not responded to standard management for non-IPF interstitial lung diseases. No recommendations were made either for or against the use of pirfenidone for the treatment of IPF [150]. The consensus avoided steroid monotherapy, combination of prednisone, azathioprine and N-acetylcysteine, N-acetylcysteine monotherapy, warfarin, therapies based on vasodilators or immunomodulators. Non-drug management procedures also include long term oxygen therapy in patients with IPF who have significant resting hypoxemia [151], pulmonary rehabilitation [152] and lung transplant based on patient preference and clinical criteria. According to the survival time of about 3 years after diagnosis, the guidelines recommend undergoing lung transplantation as close as possible to the moment of diagnosis, for those patients qualified for said surgical procedure [150]. Although lung transplantation entails a substantial risk for the patient, it has been proven that it considerably reduces the frequency of deaths associated with the disease, even more among those who survive at least one year after surgery [153].

Specifically, in the European Union, the indication of pirfenidone prior to April 2023 did not include patients with advanced IPF. However, a recent post-hoc analysis of six clinical studies revealed that clinical variables FVC and rate of all-cause mortality from baseline to week 52 were statistically different for pirfenidone compared to placebo, with no statistical differences between advanced and non-advanced IPF. In both types of IPF patients, pirfenidone showed the same safety profile. So, according to these results, the indication for pirfenidone in the European Union was extended to patients with advanced IPF [154].

### 5.2 Candidates in clinical trials

Given the preponderant role of CTGF in the development and evolution of IPF, this molecule constitutes an effective target for the development of drugs with anti-fibrotic effect. In that sense, the anti-CTGF antibody Pamrevlumab was obtained, and it works by promoting clearance of CTGF into the circulation [155]. The efficacy of this treatment was evaluated in patients with IPF in the phase 2 randomized, double blind, placebo-controlled PRAISE trial [156]. This study was conducted at 39 medical centers in seven countries in which patients received intravenous Pamrevlumab or placebo for 48 weeks. Variables examined to verify efficacy and safety included FVC, high-resolution computed tomography scans and a health-related quality of life survey. Pamrevlumab significantly reduced the decrease in FVC and the proportion of patients with disease progression compared to placebo group. The quantitative tomography scores were also significantly lower in the Pamrevlumab group, but quality of life measure at week 48 showed a non-significant improvement in the antibody group. In 2023, phase 3 ZEPHYRUS 1 (NCT03955146) clinical trial regarding the same candidate concluded, but treatment did not meet the primary endpoint of change from baseline in FVC at week 48 (p=0.29). The mean decline in FVC from baseline to week 48 was 260 ml in the pamrevlumab arm compared to 330 ml in the placebo arm (placebo-corrected difference of 70 ml; 95% CI −60 to 190 ml). Although treatment proved to be safe and well tolerated, it did not meet either the secondary endpoint of time to disease progression (FVC percent predicted decline of ≥10% or death) (HR= 0.78; 95% CI 0.52 to 1.15). According to these results, ZEPHYRUS 2 (NCT04419558) clinical trial was discontinued [157].

BI 1015550, an oral preferential inhibitor of the phosphodiesterase 4 subtype was assayed in a multicenter, randomized, double-blind, phase 2 trial (NCT04419506). Two daily 18 mg doses of BI 1015550, either alone or with background use of an antifibrotic agent, prevented a decrease in lung function in patients with IPF [158]. Based on these encouraging results, a double blind, randomized, placebo-controlled phase III (FIBRONEER-IPF, NCT05321069) was designed to test absolute change in FVC at week 52, administering 9 mg or 18 mg of BI 1015550 two times per day [159]. Another candidate, TAS-115 an oral multi-kinase inhibitor, was assayed in an exploratory phase 2 study (JapicCTI-183898). A cohort of treatment-naïve, pirfenidone, or nintedanib prescribed patients received an oral dose of 200 mg/day for 13 weeks. TAS-115 treatment met the primary endpoint, lowering the slope of the %FVC decline of 0.0750%/day at week 13. Efficacy was also demonstrated in week 26. Treatment proved to be safe and tolerable and candidate-related adverse events were mostly manageable by dose reduction, dose interruption, or symptomatic treatment [160]. Another candidate, TD139, a small-molecule inhibitor of galectin-3, was assayed in a randomized, double-blind, multicenter, placebo-controlled, phase 1/2a trial (NCT02257177). Different doses of inhaled formulation of the molecule were administered to healthy volunteers and IPF patients for 14 days. TD139 was rapidly absorbed and well tolerated with no significant treatment-related side-effects. The concentration of the molecule in the lung was >567-fold higher than in the blood, with a plasma half-life of 8 h. From an effectiveness point of view, Gal-3 expression on alveolar macrophages was reduced in the 3 and 10 mg dose groups compared with placebo, in a concentration-dependent inhibition way, and this inhibition was associated with reductions in plasma biomarkers centrally relevant to IPF pathobiology [161]. Change from baseline in FVC rate to week 26 was the primary endpoint of a multicenter, randomized, double-blind, placebo-controlled, phase 2 study (NCT01766817) with the candidate BMS-986278, a lysophosphatidic acid receptor 1 antagonist. Patients treated twice a day with the 600 mg dose experienced a significantly slower rate of decline in FVC vs placebo (p=0.049), but elevations in hepatic enzymes were observed in both BMS-986020 treatment groups [162]. Although the study was terminated early because of three cases of cholecystitis related to the candidate, some encouraging results regarding effectiveness [163, 164] promoted the design of a new phase 2, randomized, double-blind, placebo-controlled, parallel-group, international trial employing lower doses of the molecule [165].

Despite the positive results in preclinical studies, some other tested candidates did not show signs of efficacy nor safety in the clinical scenario. For example, the administration of GLPG1205, a selective functional antagonist of G-protein-coupled receptor 84, demonstrated a poorer safety and tolerability profile than placebo and, on the other hand, did not result in a significant difference in FVC decline in the phase 2, randomized, double-blind, placebo-controlled, proof-of-concept PINTA trial (NCT03725852) [166]. Another candidate, BG00011 (formerly STX-100), a humanized anti-αvβ6 IgG1 monoclonal antibody, was studied in two different clinical trials. NCT01371305 was a randomized, double-blind, placebo-controlled, dose-escalation phase 2a study, in which the primary endpoint was to test the safety and tolerability of multiple ascending doses (0.015–3.0 mg/kg, n = 8 each) of BG00011. In this case, doses less than 1.0 mg/kg were generally well tolerated, although acute IPF exacerbation occurred among patients at higher doses. Anyway, there were some signs of effectiveness like the inhibitory effect of BG00011 on pSMAD2 expression, starting at the 0.3 mg/kg dose and achieving ⩾70% reduction at 1.0 mg/kg and the diminished expression of TGF-β1 activity biomarkers [167]. These results paved the way to carry out a placebo-controlled randomized phase 2b clinical study (NCT03573505) in which the primary endpoint was the FVC change from baseline at week 26 after once-weekly subcutaneous administration of 56 mg of BG00011. At the end of the study, there was no significant difference in FVC change from baseline between patients who received BG00011 or placebo (p=0.268). Besides, IPF exacerbation/or progression was reported in 13 patients (all in the BG00011 group) and serious adverse events, including four deaths, occurred more frequently in BG00011 patients. Taking all this into account, the candidate was discontinued due to imbalance in adverse events and lack of clinical benefit [168]. Lebrikizumab, an interleukin (IL)-13 monoclonal antibody, was also assayed in a phase 2, randomized, double-blind, placebo-controlled trial, alone or with background pirfenidone therapy. The primary endpoint of the study was to establish efficacy (annualized rate of FVC % predicted decline over 52 weeks) and safety, after the subcutaneous administration of 250 mg of Lebrikizumab every 4 weeks. Although the candidate was well tolerated with a favorable safety profile, its application alone or with pirfenidone was not associated with reduced FVC % predicted decline over 52 weeks despite evidence of pharmacodynamic activity. The conclusion of this study was that blocking IL-13 may not be sufficient to achieve a lung function benefit in patients with IPF [169].

### 5.3 Experimental Anti-fibrotic Compounds

Reinforcing the relationship between hyperglycemia and pulmonary fibrosis, it has been demonstrated that different hypoglycemic agents exert positive effects in prevention of lung diseases. For example, metformin, an oral hypoglycemic drug, with strong properties as an antioxidant and anti-inflammatory molecule, attenuates lung fibrosis by inhibiting TGF-β1 signaling, modulating metabolic pathways, inducing lipogenic differentiation of fibroblasts and activating PPARG [170]. Besides, sitagliptin, a dipeptidyl peptidase 4 (DPP4) inhibitor, reduced ECM deposition and alpha SMA expression by suppressing the phosphorylation of Smad3 in lung fibroblasts stimulated with TGF-β1 in vitro [171]. Another DPP4 inhibitor, vildagliptin, has been reported to inhibit EMT, ameliorating the symptoms of fibrosis [172]. Glucagon-like peptide-1 (GLP1) and GLP1 receptor agonists, like exendin 4, have proven to decrease the expression of ECM proteins in lungs of diabetic animals and in high glucose conditions in vitro by controlling the expression of NF-kB and lowering oxidative stress [76]. On the other hand, natural and synthetic PPARG ligands have also shown antifibrotic effects [173]. PPARG agonist rosiglitazone was shown to and inhibit TGF-b1-mediated EMT and collagen synthesis in mouse models [174]. Another PPARG ligand, pioglitazone, has also shown properties as an anti-fibrotic compound in animal models [175]. Besides their anti-fibrotic effect in murine models mediated by BLM administration, PPARG ligands are also able to prevent radiation-induced pulmonary fibrosis [176]. It has been proposed that the beneficial effect of PPARG activation in controlling lung fibrosis may be due to the receptor’s broad cellular distribution within the lung, as this is expressed by alveolar epithelial cells, fibroblasts, bronchial smooth muscle cells, type II pneumocytes, macrophages, endothelial cells, lymphocytes, and dendritic cells [177].

Other substances have been tested as putative anti-fibrotic agents in vitro and in vivo, opening a wide spectrum of possibilities for the development of drugs based on components of natural origin. These elements are mainly focused on preventing the transdifferentiation of lung fibroblasts to the secretory myofibroblast phenotype, as well as on inhibiting their proliferation and migration. Among them, dihydromyricetin, a natural flavonoid extracted from vine tea, shown to restrain fibrosis in primary human and murine lung cells treated with TGF-b1 and in a bleomycin-induced mouse model of IPF by regulating the STAT3/pSTAT3/GLUT1 signaling pathway [75]. Quercetin, an antioxidant compound widely found in vegetables, fruits, tea, and wines has been shown to reduce oxidative stress and inflammatory markers in IPF by restoring senescent fibroblast sensitivity to pro-apoptotic stimuli through the activation of Akt in aged mice [178]. Other compounds such as resveratrol and indirubin also proved to repress pro-fibrotic signaling pathways and alleviate the fibrotic deposition [179,180]. Vincamine, an indole alkaloid with vasodilator properties and extracted from the leaves of Vinca minor, proved to exert an anti-apoptotic activity and attenuate the fibrotic and inflammatory conditions, suppressing EMT by modulating TGF-β1/p38 MAPK/ERK1/2/TWIST/Snai1/Slug/fibronectin/N-cadherin pathway [181].

As senescence is highly implicated in IPF development, senolytic drugs have also been assayed to target both, alveolar epithelial cells, and lung fibroblasts. These approaches are intended to act by removing senescent cells directly or by inhibiting SASP [182] and target different molecules, organelles, or molecular processes. For example, GRN510, a telomerase activator, and raloxifene, an estrogen receptor modulator molecule, were used to induce telomerase activity and maintain proper telomere size [183]. These interventions resulted in attenuation of experimental lung fibrosis, showing decreased collagen deposition and loss of lung function, and protecting lung epithelial cells from senescence. Hexafluoro, a fluorinated synthetic honokiol analogue, partly decreased TGF-β1-induced mitochondrial oxidative stress and activation of fibroblasts via sertuin-3 stimulation and diminished the levels of α-SMA and fibronectin [184]. Some other investigational studies have been conducted to modulate autophagy. Among them, rapamycin, and sphingosine 1-phosphate were assayed to activate autophagy by inhibiting mTORC1 [185]. Several elements from epigenetic mechanisms have been used as targets for inhibiting IPF. Enhanced miR 17-92 levels decrease expression of profibrotic genes including collagen 1A1 and CTGF [186]. Vorinostat, a pan-histone deacetilase (HDAC)-inhibitor, decreases lung fibrosis by promoting apoptosis of myofibroblasts, improving lung function in an experimental model in mouse [187]. Another pan-HDAC-inhibitor panobinostat decreases profibrotic phenotype and induces cell cycle arrest and apoptosis in IPF fibroblasts, more effectively than pirfenidone [188]. Another approach for inhibiting IPF is the modulation of miRNAs, by blocking pro-fibrotic miRNAs or restoring anti-fibrotic miRNAs. miR-21, miR-133a or miR-106b-5p are among the potential molecules which regulate TGF-β1 expression or function, inflammation, actin expression or cell signaling [189].

## 6. Future perspectives

The new findings regarding the molecular pathways involved in the development of IPF and DM, as well as the results obtained in clinical trials, show the need to address both medical problems from several angles at the same time, not only to slow down the progression of fibrosis in the lung and the development of other diabetic complications, but rather, to reverse the damage in peripheral organs and thereby guarantee their better functioning and higher quality of life for patients. The combination of early diagnosis and intervention, the application of combined therapies that allow the patient to be treated in a personalized way, according to the stage of their disease and their personal conditions of associated pathologies, will guarantee greater success in a short term. Essential support elements include the development of visualization technologies and quantification software, as well as biomarkers that allow accurate diagnosis and reliable monitoring of the evolution of the disease during clinical trials and in the treatment of patients with already approved drugs.
